# The Abuse of Prescriptons

**Published:** 1902-03-29

**Authors:** 


### The Abuse of Prescriptons.

It is an ancient saw of wisdom that "a man who is his own lawyer has a fool for a client," and it would be equally true if the words "doctor" and "patient" were substituted for "lawyer" and "client." But, curiously enough, as a rule, people are far more chary of risking their property by managing their own legal affairs than their health by drugging themselves. It is, indeed, astonishing with what confidence large doses of patent medicine, or of tabloids and preparations of a kindred nature, are swallowed by those who, although they may be aware of the name of the drug they are taking, cannot possibly be acquainted with its therapeutic properties or action. Unfortunately many of the more powerful drugs can easily be obtained without the necessity of a prescription from a physician; but even if the chemist refuses to supply the drug without such a warrant, it is only necessary to obtain one prescription, and it is then easy to procure any quantity. It is immaterial at what date, or under what conditions, the drug may have been prescribed; while if it be one containing opiates, and if the chemist becoming alarmed at the amount ordered refuses to supply more, it is quite easy to have the same prescription dispensed at several different establishments. In this manner the pharmacomaniac can more easily gratify his morbid appetite than the dipsomaniac, who will probably be refused alcohol by the publican when already under its influence. Undoubtedly a prescription given by a physician is the property of the patient, and we have actually heard that in the City it is not unusual for young men to sell each other copies of prescriptions which they have found efficacious in their own case. Again we all know many careful people, usually ladies, who not only keep their prescriptions, but write upon them for what condition they were given and whether the result of taking the mixture was favourable. It is, indeed, not unusual for such people to give the same medicine to their friends or servants, because it did them so much good, and we remember an unfortunate young gardener who was drugged by his mistress for two or three months with a mixture ordered for rheumatism when he was in reality suffering from quite another disorder. But besides the errors which are likely to arise in diagnosis under such conditions there is also the danger that the disease for which the drug was ordered may have been wrongly entered by the patient, as in the case of a lady who took a strong tonic, thinking it was a mixture for dyspepsia, and was puzzled why it did not do her as much good as when it was originally given. All this is most unfortunate, but as the law exists it is doubtful if there be any remedy. It is true a person is not permitted to dispose of his own life, and is heavily punished if he attempts to commit suicide and fails, for the peculiar legal reason that he is a subject of the King; but there is no law to prevent him slowly poisoning himself to death with alcohol or drugs. Now there are certain restrictions as to the sale of alcohol and also as to the dispensing of poisons, but as we have shown above anyone armed with a prescription can obtain practically as much of the drug ordered as he wishes, and it therefore is a serious question whether some restriction should not be put upon the dispensing of strong narcotics and other substances which ought only to be taken under the immediate direction and superintendence of a physician. The recent case of Forsythe *v.* Law illustrates the ease with which a patient can obtain large quantities of morphia, and if such drugs cannot safely be handled by a trained nurse, how much more dangerous must they be in the possession of the general public. Perhaps when the Pharmacy Acts are revised it would be possible to insert a clause compelling chemists to stamp upon the prescription the date when dispensed, so that others could see at a glance the amount recently supplied, or a clause forbidding the making up of mixtures containing poisons without special instructions. Most physicians are already loth to give prescriptions to people who might abuse them, or to furnish young neurotics with the means of satisfying their morbid craving for drugs, but there is no doubt that since physicians have so largely given up dispensing their own medicines in London and the large towns, a great increase in self-drugging has taken place. The argument of a young mother, who flew to the chloroform bottle whenever she had a slight headache or neuralgia, was, "But surely these things were given us to alleviate pain," and it was quite impossible to persuade her that she was not doing her duty either to herself or her children by thus risking a serious and permanent injury to her health.

